# Meteorological Register for September, 1816

**Published:** 1816-11

**Authors:** John Bailey

**Affiliations:** Surgeon, &c.


### Meteorological Register for September, 1816.

*Kept at Harwich, in Essex.*

By JOHN BAILEY, Surgeon, &c.

| Moon's Age. | | Atmospherical Pressure. Morn. | Ev. | Temp. | Wind. | Remarks. | General Statement of Diseases. |
|---|---|---|---|---|---|---|---|
| | 1 | 29 1 | 29 3 | 55 | N | Rain. Gale of w. | |
| | 2 | 5 | 6½ | 56 | N | Fine and clear | Intermittentes |
| | 3 | 7 | 6 | 55 | W | Rain | |
| | 4 | 4 | 4 | 50 | S | Much rain | Ophthalmia |
| | 5 | 5 | 7 | 59 | W | Rain | |
| ○ | 6 | 8 | 8 | 51 | NW | Foggy | Pneumonia |
| | 7 | 7½ | 7½ | 65 | NW | Fine and clear | |
| | 8 | 7 | 7 | 65 | W | ditto | Scarlatina |
| | 9 | 7 | 4½ | 60 | SW | Much rain | |
| | 10 | 5 | 7½ | 64 | W | Brisk w. Fine | Varicella |
| | 11 | 8 | 8 | 64 | W | | |
| | 12 | 9 | 9 | 65 | W | Rain | |
| | 13 | 30 1 | 30 | 60 | W | ditto | |
| ☾ | 14 | | | 68 | SW | Very fine | |
| | 15 | | | 67 | SW | ditto | |
| | 16 | 29 9½ | 29 9½ | 68 | SE | ditto | |
| | 17 | 9 | 8½ | 64 | SE | Foggy | |
| | 18 | 8½ | 30 | 59 | N | ditto | |
| | 19 | 30 | | 58 | NE | | |
| | 20 | | 29 9 | 51 | NE | | |
| ● | 21 | 29 7 | 6 | 56 | SE | Rain | |
| | 22 | 6½ | 7 | 66 | N | | |
| | 23 | 7½ | 7½ | 61 | | | |
| | 24 | 7 | 8½ | 60 | SE | Rain | |
| | 25 | 9½ | 30 ½ | 63 | SE | Foggy | |
| | 26 | 30 1 | 1 | 61 | SW | ditto | |
| | 27 | 1 | 1 | 55 | N | | |
| ☽ | 28 | | 29 9 | 61 | W | | |
| | 29 | 29 8 | 9 | 60 | W | Violent gale | |
| | 30 | 6 | 6 | 48 | W | | |

The last column of the present Report exhibits pretty much the appearance of that of last month. Some very severe cases of pulmonary disease have occurred, which have submitted, however, to the usual mode of treatment.—Pneumonia and intermittents are unusual visitants at this period of the year; but the extraordinary wetness of the time, accompanied by the low degree of atmospheric temperature, have given the feel and appearance of February; and along with it, the disorders peculiar to that season. There has not fallen within the observation of this Reporter *one* occurrence of autumnal bilious diarrhœa. Wet and disagreeable as the whole summer has proved, it has not been so sickly as in common.

